# Iris neovascularization and neurotrophic keratopathy following ultrasound cycloplasty in refractory glaucoma: case series

**DOI:** 10.1186/s12886-024-03460-3

**Published:** 2024-04-23

**Authors:** Zidong Chen, Yanyan Wu, Minbin Yu

**Affiliations:** State Key Laboratory of Ophthalmology, Zhongshan Ophthalmic Center, Sun Yat-Sen University, Guangzhou, China

**Keywords:** Ultrasound cycloplasty, Refractory Glaucoma, Iris neovascularization, Neurotrophic keratopathy

## Abstract

**Background:**

Ultrasound cycloplasty is a noninvasive surgery used to reduce intraocular pressure in patients with glaucoma, with fewer severe complications. This report presents several cases of iris neovascularization and neurotrophic keratopathy following ultrasound cycloplasty.

**Case presentation:**

Six patients diagnosed with refractory glaucoma underwent ultrasound cycloplasty at our clinic. Three cases developed iris neovascularization at postoperative day 3, week 2 and week 4 respectively, with intraocular pressure ranging from 12 to 24 mmHg. The other three cases developed neurotrophic keratopathy at postoperative week 3, week 6 and week 8 which completely healed within 60 days.

**Conclusions:**

Iris neovascularization and neurotrophic keratopathy can be triggered after ultrasound cycloplasty, which are uncommon and self-limited but potentially vision-threatening. Preoperative risk assessment and regular postoperative follow-up are recommended to manage complications effectively.

## Background

Ultrasound cycloplasty (UCP) is a recently developed procedure that uses miniaturized transducers to produce high-intensity focused ultrasound cyclocoagulation of the ciliary body [1]. It is a noninvasive glaucoma procedure that permits a selective and controlled thermic effect on the distal part of the ciliary body with limited damage to adjacent structures [2, 3]. UCP decreases intraocular pressure (IOP) effectively by reducing aqueous humor production and increasing uveoscleral outflow [2, 4]. Compared to other glaucoma surgeries, UCP has shown a lower rate of intraoperative or postoperative complications and is increasingly used in treatment-naive glaucoma patients with better visual acuity [5]. Reported complications of UCP include conjunctival hyperemia, anterior chamber inflammation, superficial punctate keratitis, corneal edema, subconjunctival hemorrhage, superficial corneal ulceration, transient IOP spike, loss of visual acuity, hypotonia and macular edema [4]. This case series discusses the development of neovascularization of the iris (NVI) or neurotrophic keratopathy (NK) following UCP. To our knowledge, NVI post UCP has not been previously described in the literature, and the precise mechanism of superficial corneal ulceration post-UCP remains unknown.

### Case presentation

To date, we have performed over 600 UCP operations at our clinic. We analyzed the clinical data of the six patients who developed NVI (3 patients, less than 0.7%) or NK (3 patients, less than 0.6%) following UCP (Table 1). These complications were found to be rare. The surgical technique involved UCP with paracentesis using standard parameters (10 sectors, 21 MHz of frequency, 8 s ultrasound, 20 s pause, 225 mmHg external aspiration). Postoperatively, topical antibiotic combined with dexamethasone were prescribed as per routine clinical practice and gradually tapered off within one month.

**Table 1:** Summary of the six case reports

Case	1	2	3	4	5	6
Diagnosis	PACG	PACG	PACG	Secondary glaucoma	Secondary glaucoma	Juvenile glaucoma
Age	37	35	64	14	21	34
Ocular operation history	4	2	1	3	2	4
Pre-UCP VA	20/40	20/200	20/200	20/200	20/40	20/40
Pre-UCP IOP(mmHg)	45	32	45	38	17	35
IOP-lowering drops	4	3	3	4	4	4
1 day post-UCP VA	20/40	20/200	20/200	20/200	20/200	20/50
1 day post-UCP IOP(mmHg)	35	25	15	24	22	20
Time of NVI occurred(post-UCP)	Week 4	Day 3	Week 4	-	-	-
Time of NVI disappeared(post-UCP)	Unknown	Week 8	Week 6	-	-	-
Time of Corneal ulceration occurred(post-UCP)	-	-	-	Week 6	Week 3	Week 8
Time of Corneal ulceration healed(post-UCP)	-	-	-	Week 14	Week 5	Week 12

*PACG* primary angle closure glaucoma, *UCP* ultrasound cycloplasty, *VA* visual acuity, *IOP* intraocular pressure, *NVI* neovascularization of the iris

### Case 1

**Fig. 1:**
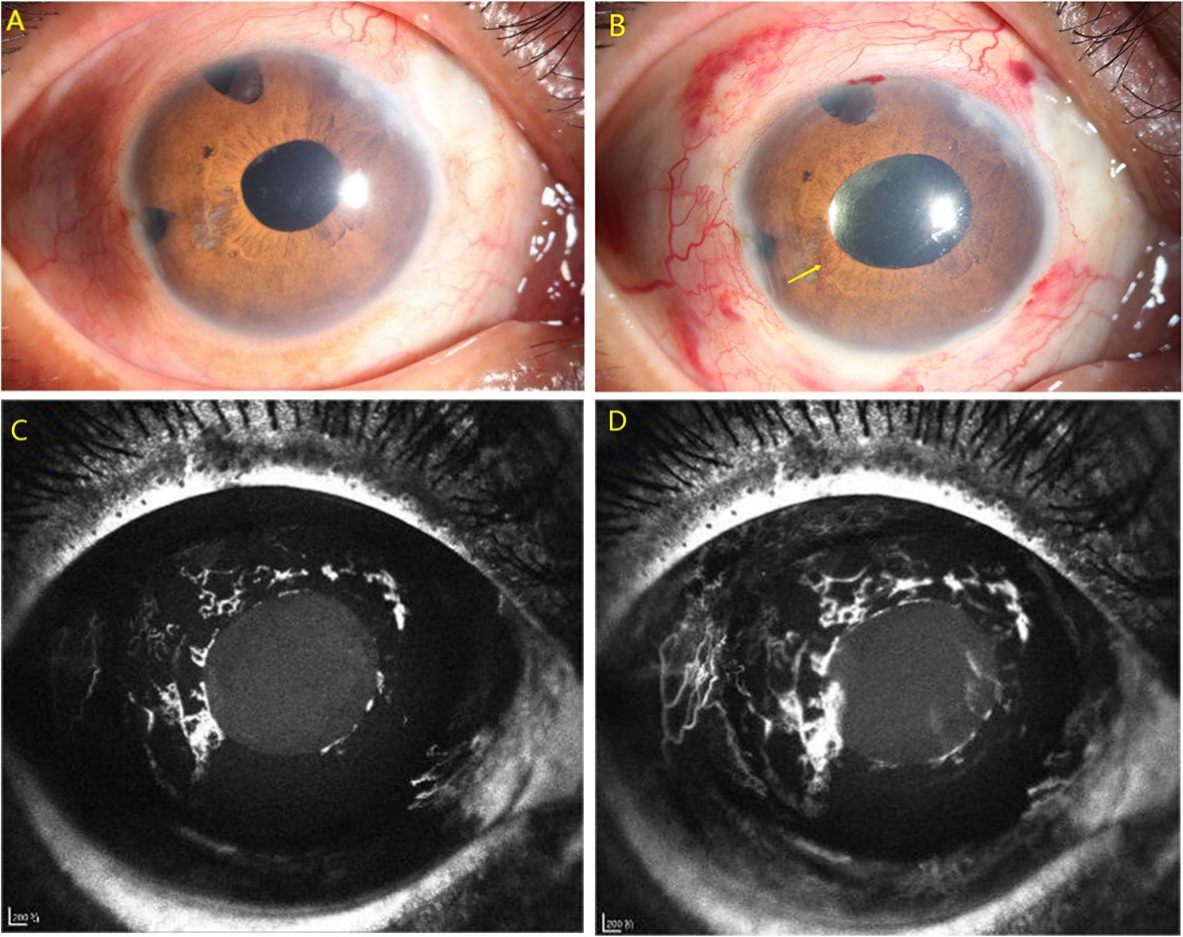
Preoperative slit-lamp photograph of the right eye in case 1 (**A**). Slit-lamp photographs showed a mild dilated pupil and 270 degrees of neovascularization of the iris (**B**, NVI was labeled by yellow arrow). Iris fluorescein angiography revealed neovessel with early dye leakage around the pupil (**C**,**D**)

**Fig. 2:**
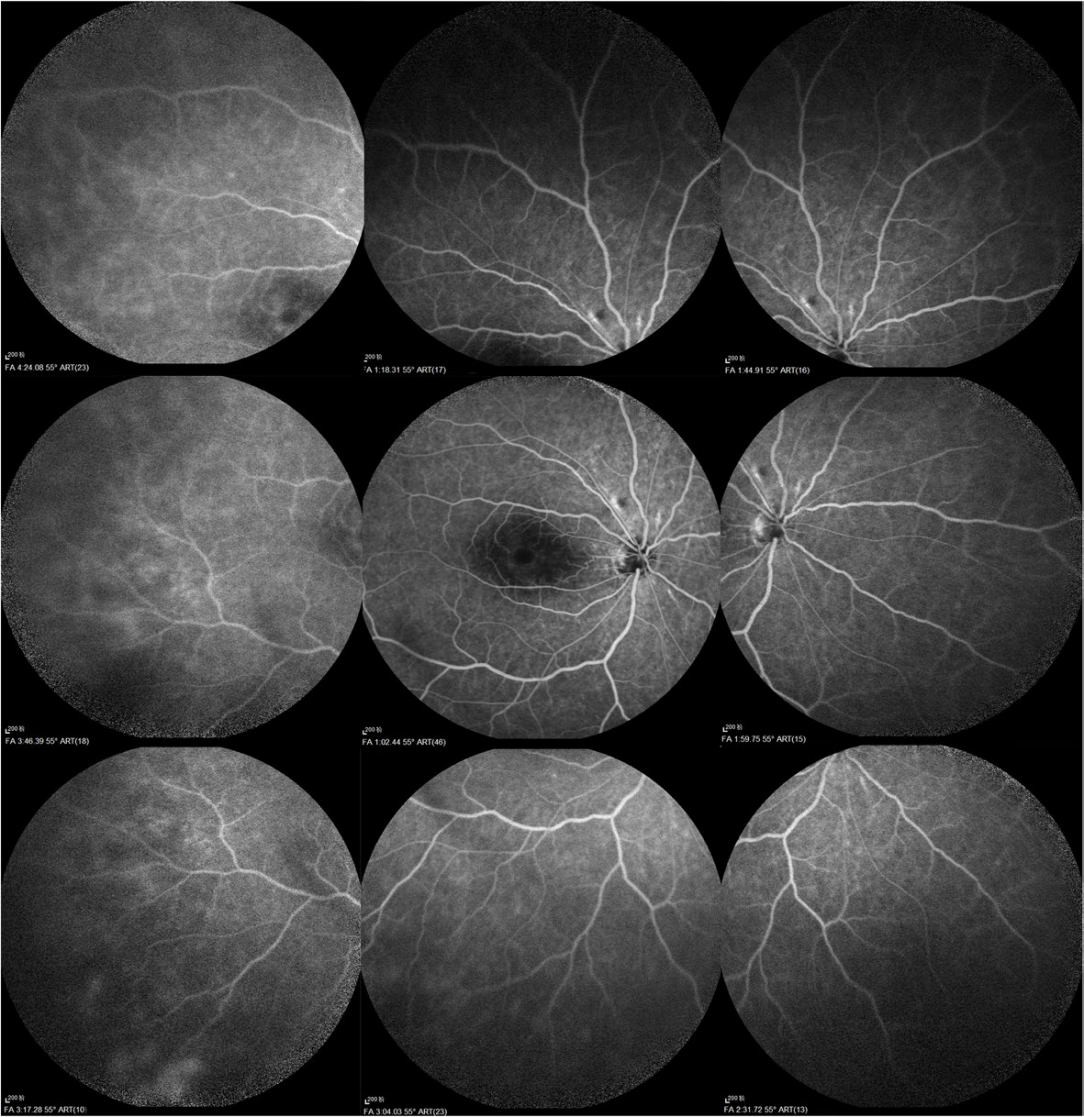
Fundus fluorescein angiography of the right eye in case 1 showed no sign of posterior segment ischemia

A 37-year-old Chinese male diagnosed with primary angle closure glaucoma underwent UCP with paracentesis in the right eye. His ocular history included three trabeculectomy and cataract surgery. At presentation (Fig. 1A), the visual acuity (VA) was 20/40 and IOP was 45 mmHg. At the second postoperative week, slit-lamp examination revealed a mild dilated pupil and 270 degrees NVI (Fig. 1B), with VA of 20/200 and IOP of 12 mmHg. Iris fluorescein angiography showed the formation of neovessels with early dye leakage around the pupil (Fig. 1C, D). Fundus fluorescein angiography showed no sign of posterior segment ischemia (Fig. 2). The patient was prescribed systemic and topical glucocorticosteroid but returned to local hospital for the further follow-up due to financial constraints.

### Case 2

**Fig. 3:**
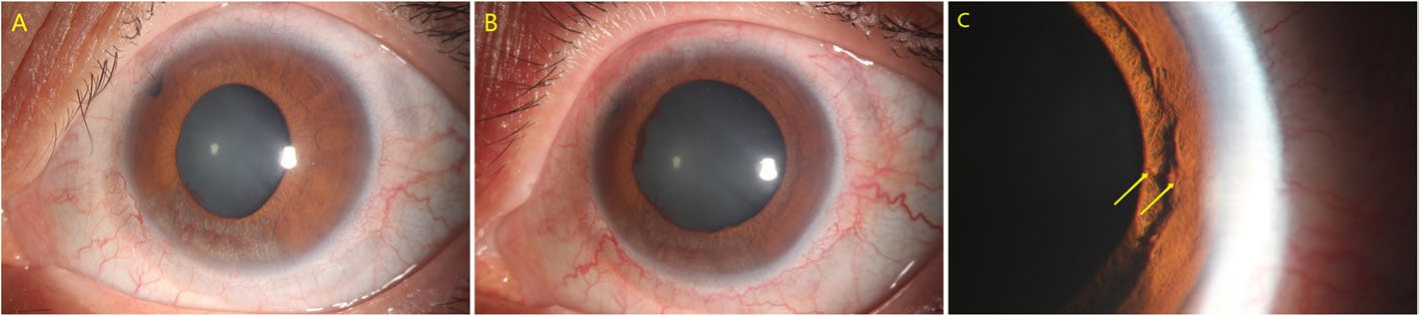
Preoperative slit-lamp photograph of the left eye in case 2 (**A**). Slit-lamp photographs revealed a mild dilated pupil (**B**) and neovascularization of the iris (**C**,NVI was labeled by yellow arrows)

A 35-year-old Chinese female diagnosed with angle closure glaucoma secondary to autosomal recessive bestrophinopathy underwent UCP with paracentesis in the left eye. Her ocular history included peripheral iridotomy and a previous UCP procedure. At presentation (Fig. 3A), her VA was 20/200 and IOP was 32 mmHg. Three days after the surgery, slit-lamp examination revealed a mild dilated pupil and NVI in left eye (Fig. 3B, C), with VA of 20/200 and IOP of 24 mmHg. Oral and topical nonsteroidal anti-inflammatory medications were prescribed. The NVI finally disappeared within two months.

### Case 3

**Fig. 4:**
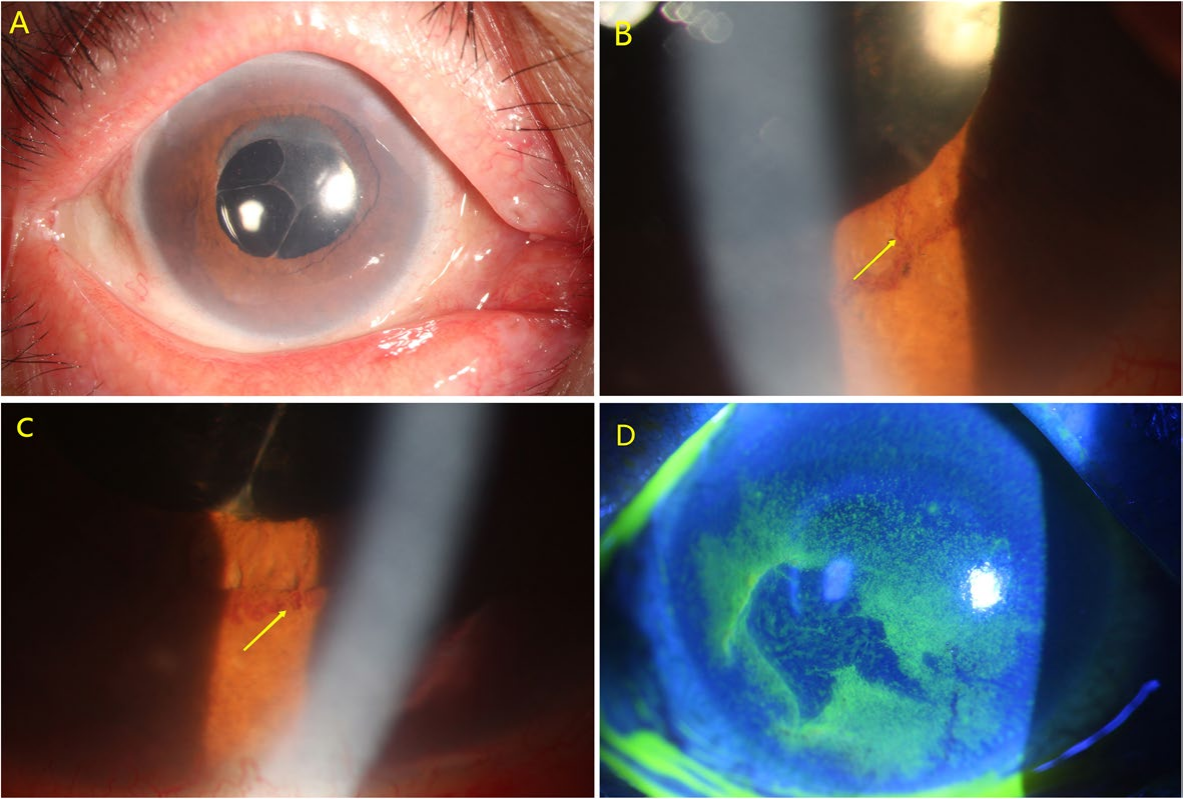
Preoperative slit-lamp photograph of the right eye in case 3 (**A**).Slit-lamp photographs revealed neovascularization of the iris (**B**,**C**,NVI was labeled by yellow arrows) and a mild corneal epithelial defect (**D**)

A 64-year-old Chinese female diagnosed with primary angle closure glaucoma underwent UCP with paracentesis in the right eye. Her ocular history included combined phacotrabeculectomy surgery. At presentation (Fig. 4A), her VA was 20/200 and IOP was 45 mmHg. At the forth postoperative week, a 90-degree NVI and a mild corneal epithelial defect were noticed in right eye (Fig. 4B, C, D). Examination revealed VA of 20/200 and IOP of 17 mmHg. Topical glucocorticosteroid and healing drops containing deproteinized calf blood extract, recombinant human epidermal growth factor, and recombinant bovine basic fibroblast growth factor were administered. The corneal epithelial defect healed within one week. And the NVI eventually disappeared within two weeks.

### Case 4

**Fig. 5:**
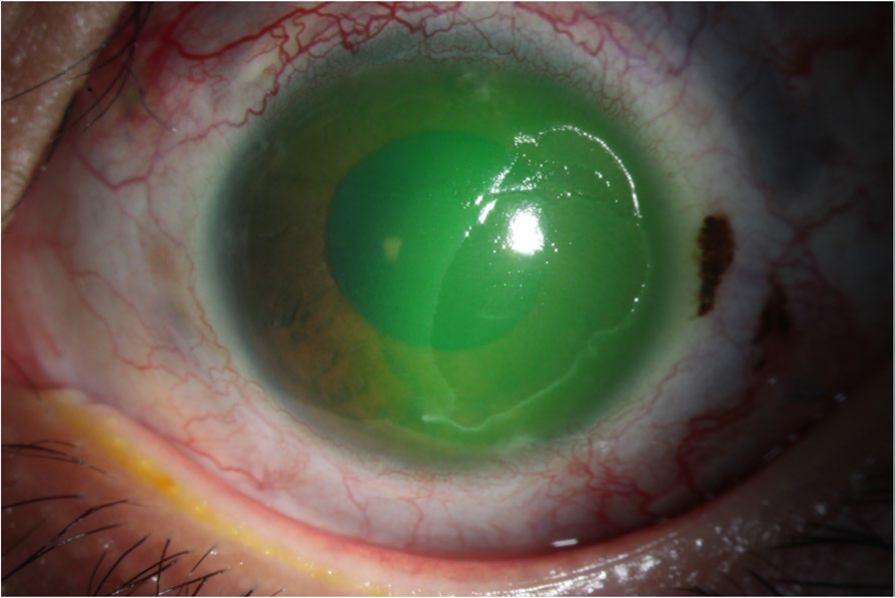
Slit-lamp photograph revealed a temporal corneal epithelial defect measuring 7 mm vertical × 4 mm horizontal in left eye

A 14-year-old Chinese boy diagnosed with glaucoma secondary to silicone oil injection in left eye underwent UCP wtih paracentesis. His ocular history included scleral encircling operation, trabeculectomy and vitrectomy combined with silicone oil injection. Preoperatively, his VA was 20/200 and IOP was 38 mmHg. At the sixth postoperative week, the patient presented with a painless temporal corneal epithelial defect measuring 7 mm vertical × 4 mm horizontal in left eye (Fig. 5). Corneal sensation was reduced in all quadrants, tested with a cotton-tipped applicator. The VA was 20/100 with IOP of 16 mmHg. Topical healing drops and contact bandage lens were prescribed. The corneal defect completely resolved within 60 days.

### Case 5

**Fig. 6:**
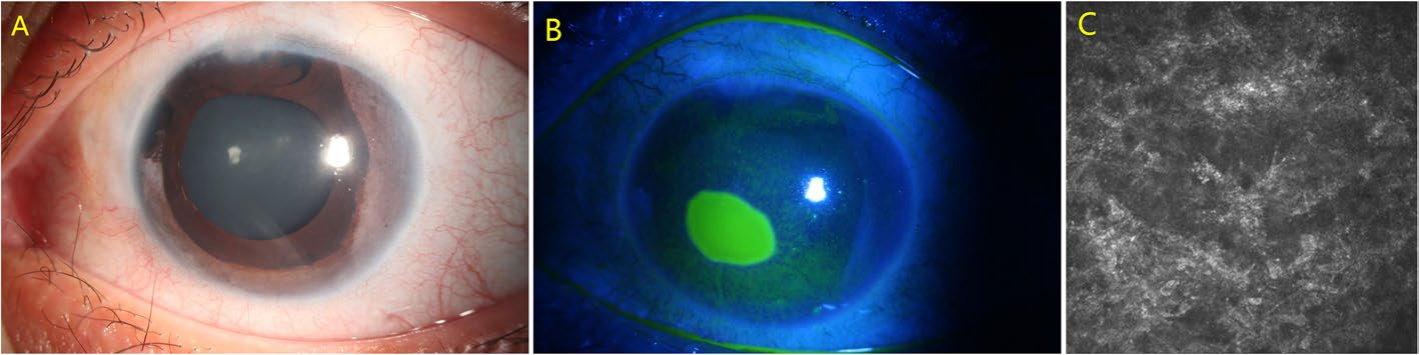
Preoperative slit-lamp photograph of the left eye in case 5 (**A**). Slit-lamp photograph revealed a nasal corneal epithelial defect measuring 2.5 mm vertical × 3 mm horizontal in left eye (**B**). Confocal microscopy revealed no subepithelial nerve fiber plexus seen under the defect area (**C**)

A 21-year-old Chinese female diagnosed with glaucoma secondary to congenital ectropion uvea underwent UCP with paracentesis in left eye. Her ocular history included two trabeculectomy procedures. Preoperatively, her VA was 20/40 and IOP was 17 mmHg (Fig. 6A). At the third postoperative week, a painless nasal corneal epithelial defect measuring 2.5 mm vertical × 3 mm horizontal was noticed in left eye, with VA of 20/200 and IOP of 8 mmHg (Fig. 6B). Corneal sensation was reduced in all quadrants, as tested with a cotton-tipped applicator. Confocal microscopy revealed no subepithelial nerve fiber plexus under the defect area (Fig. 6C). The corneal defect almost resolved within 15 days with topical treatments.

### Case 6

**Fig. 7:**
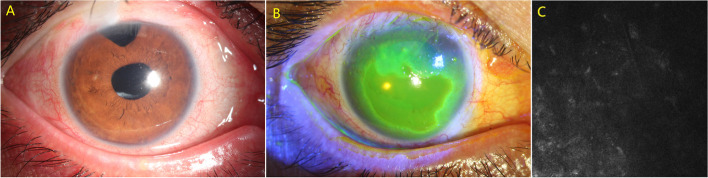
Preoperative slit-lamp photograph of the right eye in case 6 (**A**). Slit-lamp photograph revealed a central corneal epithelial defect measuring 5 mm vertical × 7 mm horizontal in the right eye (**B**). Confocal microscopy revealed no subepithelial nerve fiber plexus (**C**)

A 34-year-old Chinese male diagnosed with juvenile glaucoma underwent UCP with paracentesis in right eye. His ocular history included trabeculectomy, cataract surgery, vitrectomy combined with silicone oil injection, and subsequent silicone oil removal. At presentation (Fig. 7A), his VA was 20/40 and IOP was 35 mmHg. At the 8-week postoperative visit, examination revealed a painless central corneal epithelial defect measuring 5 mm vertical × 7 mm horizontal in right eye (Fig. 7B). Corneal sensation was reduced in all quadrants, as tested with a cotton-tipped applicator. Confocal microscopy examination revealed no subepithelial nerve fiber plexus (Fig. 7C). The VA was 20/50 with IOP of 16 mmHg. The corneal defect healed within 30 days with topical treatments.

## Discussion

UCP is a method developed more recently that uses high-intensity focused ultrasound to partially destroy the ciliary body for the management of glaucoma. This procedure is believed to reduce IOP by 30%-35%, with mild postoperative inflammation and well tolerance [2, 6, 7]. However, our observations suggest that UCP may induce the development of NVI and NK.

NVI is mostly attributed to ocular ischemic conditions. Posterior segment ischemia, such as retinal vein occlusion and diabetic retinopathy, is the most common cause of anterior segment neovascularization. The occurrence of NVI is a sign of extreme retinal ischemia. However, NVI can also manifest after various ocular surgeries as a result of anterior segment ischemia (ASI). Ocular ischemic syndrome is another causes of NVI. And the severity of ASI can vary from mild to vision threatening. The link between ASI and NVI has been documented in clinical case reports and animal model [8–11].

The anterior segment receives its blood supply from the anterior ciliary arteries and the long posterior ciliary arteries. Surgical procedures that damage the anterior ciliary arteries, which provide 70% of the anterior blood supply, are the main cause of ASI [12]. Strabismus surgery and circular buckling surgery can lead to ASI, and although it is less common, this complication can occur after uneventful anterior segment surgeries such as trabeculectomy and pterygium surgery [9, 13, 14]. Ciliary body ablation surgery, such as cyclocryotherapy and cyclophotocoagulation, which aim to destroy the function of pars plicata, carry a high risk of ASI [15]. Pathophysiology studies have demonstrated vessel necrosis of the ciliary body and subsequent neovascularization [16]. In our case series, three patients developed NVI after UCP. Preoperative slit-lamp examination and indirect ophthalmoscopy examination revealed clear iris texture and normal fundus, with no systematic diseases. Although high IOP in advanced glaucoma patients can impair ocular blood supplement, UCP was considered an inducing or aggravating factor of ASI that led to NVI. Additionally, the use of 10 sectors of treatment range in UCP may worsen ASI.

Superficial corneal ulceration following UCP has been previously reported, primarily in elderly patients with pre-existing corneal disease and a history of multiple topical treatments [17–19]. This corneal complication typically resolved within 30 days, either spontaneously or with topical treatment. In our case series, three patients developed painless corneal epithelial defects after UCP, with confocal microscopy revealing no subepithelial nerve fiber plexus in the defect area, which could be diagnosed as NK. NK has been described as a corneal complication following transscleral cyclophotocoagulation and cyclocryotherapy and is characterized by reduced corneal sensitivity, spontaneous epithelial breakdown and impaired corneal healing, despite frequent lubrication [20, 21]. All patients in our cases series had a history of chronic use of topical anti-glaucoma medications and prior ocular surgeries, but they did not develop superficial corneal defects prior to UCP treatment. Preoperative slit-lamp examination revealed clear corneas in all patients. These findings suggest that UCP may contribute to the development of NK. Furthermore, it may increase the susceptibility of patients to infectious corneal ulcerations.

NK following transscleral cyclophotocoagulation is believed to result from laser damage to the long ciliary nerves, which are responsible for sensation and play a significant role in the blink reflex as well as the integrity and function of the corneal epithelium [22]. UCP raises tissue temperature up to 80℃, leading to coagulation necrosis of the ciliary body epithelium. Despite efforts to spare the nasal and temporal zones during UCP treatment, where the long ciliary nerves innervate the anterior segment, three patients in our report still developed NK. This suggests that the long ciliary nerves may be thermally damaged during UCP treatment.

Considering the similar mechanisms to other cyclodestructive surgeries, the potential of UCP to induce anterior segment ischemia and damage the ciliary nerves should not be overlooked. In addition to the frequently reported complication of pupil abnormalities, a mechanism similar to Urrets-Zavalia syndrome may exist in the early postoperative period following UCP [23, 24]. Despite the current design of probe placement avoiding the horizontal meridian to minimize disturbance of the long ciliary nerves, surgeons must still exercise caution. Furthermore, Hayreh et al. demonstrated that involving the vertical rectus muscles results in more severe ASI symptoms, suggesting that the vertical meridian should also be spared during UCP [25].

## Conclusion

Our cases reports demonstrate that NVI and NK might occur as complications following UCP, although they are rare and self-limited. Nonetheless, they can potentially vision threatening. Further investigation into the optimal probe location and iris perfusion using confocal microscopy and iris fluorescein angiography are wanted.

## Data Availability

The datasets used and/or analysed during the current study are available from the corresponding author on reasonable request.

## Abbreviations

UCP: Ultrasound cycloplasty
IOP: Intraocular pressure
NVI: Neovascularization of the iris
NK: Neurotrophic keratopathy
VA: Visual acuity
ASI: Anterior segment ischemia
